# Evolution of the Therapeutic Management of Giant Cell Arteritis: Analysis of Real-Life Practices over Two Timeframes (2014–2017 and 2018–2020)

**DOI:** 10.3390/jcm12227105

**Published:** 2023-11-15

**Authors:** Hubert de Boysson, Anael Dumont, Paul Castan, Sophie Gallou, Jonathan Boutemy, Gwénola Maigné, Nicolas Martin Silva, Alexandre Nguyen, Samuel Deshayes, Achille Aouba

**Affiliations:** 1 Department of Internal Medicine, Caen University Hospital, 14000 Caen, France; dumont-an@chu-caen.fr (A.D.); gallou-s@chu-caen.fr (S.G.);; 2 UFR de Santé, University of Caen Normandie, 14000 Caen, France

**Keywords:** giant cell arteritis, glucocorticoids, tapering, therapeutic strategy

## Abstract

**Objectives:** To determine how therapeutic strategies for giant cell arteritis (GCA), especially glucocorticoid (GC) management, evolved between 2014 and 2020. **Patients and Methods:** Consecutive GCA patients followed for at least 24 months in a single tertiary center were enrolled and separated into two groups: those diagnosed from 2014 to 2017 and those diagnosed from 2018 to 2020. GC doses (mg/kg/day) were analyzed at onset, at Month 3 (M3) and, if continued, at M6, M12, M18 and M24. Physicians’ practices were also individually analyzed. **Results:** Among the 180 patients included, 96 (53%) were diagnosed in 2014–2017 and 84 (47%) in 2018–2020. All patients received GC at diagnosis without a difference in the initial dose between the two groups (*p* = 0.07). At M3, the daily dose was lower in patients treated after 2017 (*p* = 0.002). In patients who still received GC at M6 (*p* = 0.0008), M12 (*p* = 0.01) and M24 (*p* = 0.02), the daily GC dose was still lower in patients treated after 2017. The proportion of patients who definitively discontinued GC use before M18 (42% versus 21%, *p* = 0.003) was higher in those treated after 2017. The rates of immunosuppressant use were not different between the two time periods (31% versus 38%, *p* = 0.34), but tocilizumab replaced methotrexate. Significant differences were observed among practitioners regarding the GC doses at M6 (*p* = 0.04) and M12 (*p* = 0.04), the total GC duration (*p* = 0.02) and the ability to stop GC before M18 (*p* = 0.007). **Conclusions:** This real-life study showed a global change in GC management over time for GCA patients, with important variability among physicians’ practices.

## 1. Introduction

Giant-cell arteritis (GCA) is the most frequent systemic vasculitis in patients over 50, with a mean age around 70. Women are twice as likely as men to be affected. Although its pathophysiology remains partially understood, the disease mainly affects the branches of the external carotid, especially the temporal arteries. This vascular involvement explains the cephalic symptoms observed in more than 80% of patients, including headaches, scalp tenderness, or jaw claudication. In addition, in half of patients, GCA can also affect the aorta and its main branches, leading to aortitis and/or large-vessel stenosis. Since large-vessel vasculitis is often non-symptomatic at the initial phase, imaging (aorta CT angiography, positron emission tomography coupled with a CT scan (FDG-PET/CT), or aorta magnetic resonance angiography (MRA)) is required to determine whether the aorta and its main branches are involved. Diagnosis of GCA relies on the demonstration of the vasculitis process, either on histology, especially on a temporal artery biopsy (TAB), or by demonstrating the vascular inflammation on temporal and/or large-vessel imaging. At the initial phase of the disease, the risk of ischemic events, especially ophthalmologic and cerebrovascular, is high and warrants a rapid introduction of treatments [1].

Since the 1950s, glucocorticoids (GC) have been the therapeutic cornerstone for giant cell arteritis (GCA) [2,3,4]. No treatment has shown a better and prompter efficiency in controlling both systemic and vascular inflammation at the initial phase of the disease, resulting in a drastic reduction in ischemic complications. International guidelines still recommend using daily GC as a first-line treatment [2,3,5,6]. French guidelines at the time of this study recommended starting GC at the dose of 1 mg/kg/day in GCA patients with ischemic complications, eventually preceded by intravenous methylprednisolone pulses. The beneficial effect of immunosuppressants in these patients is not proven and actually not recommended. Otherwise, in non-complicated GCA, a starting GC dose of 0.7 mg/kg/day is recommended [5]. In accordance with current guidelines, all patients with GCA in France are treated with GC alone and this treatment is often sufficient. The use of tocilizumab (TCZ) in combination with GC at GCA diagnosis is actually limited to certain situations and should be discussed with GCA experts (e.g., relapsing patients, patients with high cardiovascular risk, or with past osteoporotic fractures or complicated diabetes mellitus). French or English recommendations as well as the 2018 European guidelines advised maintaining daily GC use for at least 18–24 months given the high risk of relapse, affecting nearly half of patients [2,5,6]. In addition, a few studies have demonstrated that alternative regimens, such as GC intake every other day or shorter treatment durations, lead to higher relapse rates [7,8]. Conversely, since the early 2000s, awareness of GC-related toxicity has emerged, especially in the GCA population, which often includes older individuals [9,10,11,12,13].

Therefore, the concept of GC-sparing strategies was born, and efforts were made to leverage therapeutic strategies to reduce GC exposure [3,14]. The addition of immunosuppressants to GC was studied, and many immunosuppressants failed to demonstrate a significant GC-sparing effect [15,16]. Among those first tried, only methotrexate may have shown a slight and inconstant beneficial effect in a metanalysis [17]. More recently, changes occurred in 2016 and 2017 with the validation of TCZ use, an anti-interleukin 6 receptor antibody, which showed a significant GC-sparing effect [14,18]. In the Giacta study, the 3-year analysis showed that patients who received TCZ had twofold smaller GC cumulative doses than those who received GC alone [19]. As a consequence, the 2021 American College of Rheumatology recommendations broke a 70-year lasting paradigm in GCA treatment by proposing for the first time the use of TCZ in combination with GC at GCA diagnosis and recommending GC withdrawal as soon as possible [3].

In recent years, some French and European studies have indicated that GCA patients received GC for a duration between 18 and 24 months [11,20,21,22]. Real-life data about GC toxicity also emphasized the high prevalence of GC-related adverse events in these patients [10,11,12,13,23,24]. Taken together, the improved knowledge of early and late GC toxicity along with the availability of GC-sparing treatments are probably changing GCA management. In this single-center study based on a collective and individual analysis of physicians’ practices over a 7-year duration (2014–2020), we aimed to analyze whether the therapeutic management of GCA changed, especially regarding the use of GC and immunosuppressants. We especially analyzed whether the approval of TCZ as standard treatment for GCA allowed for a reduction in GC exposure.

## 2. Patients and Methods

### 2.1. Patient Selection

Since 2000, data for all consecutive patients diagnosed with GCA at our tertiary center in a northwestern French region have been centralized in a database. The cohort was described in previous studies [25]. For the purpose of this study, we included all patients diagnosed with GCA who were followed up in our department from January 2014 to 31 December 2020. All patients had a follow-up duration of at least 24 months. We excluded (1) patients who died within 24 months postdiagnosis; (2) patients who were not seen for medical control at least ≥24 months after diagnosis; and (3) patients who were lost to follow up within the first 24 months.

Given the first validations of TCZ in 2016–2017 [14,18], for the purpose of this study, patients were separated into two groups: those diagnosed from 2014 to 2017 versus those diagnosed from 2018 to 2020.

This research was carried out in agreement with the ethical principles derived from the Declaration of Helsinki and good clinical practice. At the time of the study, in accordance with French law (article L1121-1-1 and article L1121-1-2), formal approval from an ethics committee was not required for this type of study.

### 2.2. Studied Parameters and Definitions

The centralized database included information regarding demographics, cardiovascular risk factors, clinical manifestations at onset, laboratory tests, histology and imaging results when available, treatments administered and outcomes including relapses, the occurrence of cardiovascular events, or death. Regarding treatments, GC management was detailed, and each dose was given in mg/kg/day of a prednisone equivalent. GC doses were recorded at initiation, at Month 3 (M3) and, when continued, at M6, M12, M18 and M24. For each patient, the GC duration was noted until discontinuation or the last follow-up visit. Patients for whom we noted GC was discontinued did not relapse after GC withdrawal. We eventually noted patients who were able to stop GC before M12, M18 and M24. Finally, we recorded whether patients received an adjunctive immunosuppressant and the chosen molecule.

Relapse was defined by the recurrence of GCA symptoms and increased acute phase reactants in a patient that previously was in remission and that responded to treatment increase. Eventually, in some patients, an isolated increase in inflammatory markers defined a relapse only if no other cause than GCA was identified and if inflammatory parameters improved with treatment increase. In patients under TCZ, since inflammatory parameters are less prone to change with disease activity, relapse was defined by clinical symptoms and, if available, by imaging.

GCA presentations and management in the two timeframes were compared. To analyze the individual practices in our department, we separated patients according to their treating physicians. Among the 7 physicians from our department who participated in this study, 5 have been following GCA patients since 2014 and 2 have been following GCA patients since 2017. In addition, among the 5 more experienced physicians, 3 followed enough patients in the time period to distinguish between the two timeframes (2014–2017 and 2018–2020), allowing for comparisons of practices.

### 2.3. Statistical Analyses

Categorical variables are expressed as the number (%), and quantitative variables are expressed as the median [range]. Categorical variables were analyzed using the chi-square or Fisher’s exact test, and quantitative variables were analyzed using the Wilcoxon rank-sum test. The statistical analyses were computed using JMP 9.0.1 (SAS Institute Inc., Cary, NC, USA). A *p* ≤ 0.05 defined statistical significance.

## 3. Results

### 3.1. Patients’ Descriptions

Between January 2014 and 31 December 2020, 198 patients were diagnosed with GCA and followed up in our department. We excluded 18 patients: 14 who died within the first 24 months and 4 who did not have at least one medical visit at ≥24 months. Among the 180 included patients, 96 (53%) were diagnosed and followed up between 2014 and 2017, and 84 (47%) were diagnosed and followed up between 2018 and 2020. The baseline characteristics of the patients are detailed and compared in Table 1. When compared to those diagnosed in the 2014–2017 period, GCA patients diagnosed after 2017 had a higher body weight (*p* = 0.02) and more frequently underwent large-vessel imaging within the first 15 days following treatment initiation (*p* = 0.04). However, the rate of large-vessel vasculitis on imaging was not different. The initial presentation and paraclinical workup did not differ between the two periods.

### 3.2. Comparison of Treatments and Outcomes during the Two Timeframes

Data about treatments and outcomes are included in Table 1. All patients received GC at GCA diagnosis without a difference in the initial daily dose in the two groups (*p* = 0.07). At M3, the 180 patients still received GC and the daily dose was lower in patients treated after 2017 (*p* = 0.002). In patients who still received GC at M6 (*p* = 0.0008), M12 (*p* = 0.01) and M24 (*p* = 0.02), the daily GC dose was still lower in patients treated after 2017. The proportion of patients who were able to definitively discontinue GC use before M12 (15% versus 5%, *p* = 0.03), M18 (42% versus 21%, *p* = 0.003) and M24 (57% versus 41%, *p* = 0.03) was higher in the group treated after 2017. The rates of immunosuppressant use were not different between the two time periods (31% versus 38%, *p* = 0.34), but methotrexate use diminished over time (*p* = 0.04), whereas TCZ use increased (*p* = 0.04).

In comparison with patients who did not receive TCZ, more patients with TCZ were able to stop GC use before M12 (26% versus 7%, *p* = 0.004), but this difference was not observed at M18 (39% versus 29%, *p* = 0.28). No patient under methotrexate treatment during both periods was able to stop GC before M12, and a total of 3/31 patients (10%) were able to stop GC before M18.

At the last follow-up visit, the relapse and death rates were not different between the two groups.

### 3.3. Evolution of the Practices of the Different Physicians

The main differences in the physicians’ practices are noted in Table 2. Significant differences were observed among the seven practitioners regarding the performance of large-vessel imaging within the first 15 days of treatment (*p* = 0.04), the GC doses at M6 (*p* = 0.04) and M12 (*p* = 0.04), the total duration of GC use in patients who stopped GC (*p* = 0.02) and the ability to stop GC before M18 (*p* = 0.007).

Individual analyses are shown in Table 3. When analyzing the three physicians who had followed patients since 2014 and for whom the two time periods were analyzable, two significantly reduced their duration of GC use (P1 and P3). The age or experience of the physicians did not influence GC management.

## 4. Discussion

This real-life study captured how the practices of GCA management evolved in a tertiary center with experience in this type of vasculitis. Regarding the general management of our GCA patients, we observed a reduction in GC exposure and a progressive increase in TCZ use over time, without differences regarding outcomes, especially the relapse rate. Importantly, most observational studies, including ours, have indicated that the total duration of GC use in GCA patients often exceeds 18 months [22]; this study indicated that >40% of patients treated after 2017 were able to discontinue GC before 18 months and 15% were able to discontinue GC before M12.

The reduction in GC exposure is probably linked to two non-independent changes in our medical practices. First, since the Giacta study [14], faster GC tapering schedules have been used, and GC doses are currently frequently decreased to less than 5 mg/day of prednisone equivalent until withdrawal. Before 2017, as frequently done in other systemic vasculitides, a maintenance dose of 5 mg/day was often prescribed for some GCA patients, prolonging the total intake duration. Second, since the good GC-sparing effect of TCZ was demonstrated in the Giacta trial, the use of an immunosuppressant in GC-dependent or relapsing GCA patients is initiated early, allowing for faster GC tapering [3].

The risk of visual loss is important at the initial phase of the disease and warrants the rapid introduction of high GC doses. Thanks to the progress observed in the two last decades in the early identification of GCA, the frequency of visual ischemic complications declined in some population-based studies [26]. Over the 7-year period of this study, we did not observe a significant reduction in GCA-related ophthalmologic involvement. However, we showed that the reduction in GC duration was not associated with an increase in ophthalmologic events nor relapse rates.

Interestingly, when analyzing individual practices in this study, significant discrepancies regarding GC management were exhibited, irrespective of the age or experience of the treating physician. Some physicians in our center drastically changed their practices and had a twofold reduction in their GC duration, whereas others did not significantly change their practices over time. Since we observed important variations among a single-center experienced team, this point emphasizes how delicate the interpretations of large and multicenter observational studies reporting treatments and outcomes might be. This also more extensively highlights how different GCA management can be in a real-life setting in comparison with the therapeutic schedules imposed in therapeutic trials. In addition to guidelines, the treatment of GCA still mainly relies on personal practices and experiences. The duration of GC use (less or more than 12 months) or the choice of the immunosuppressant and the time of its introduction between GCA diagnosis and relapse are still matters of debate among GCA experts, which is particularly highlighted in our study.

This study showed the following notable strengths: a detailed analysis of GC management in a real-life setting, including the dose in mg/kg, the mandatory follow-up time and the distinction of practices according to the different physicians. However, the retrospective and observational nature of this study is a limitation. Although the rate of ophthalmologic signs or the GC starting dose did not change between the two timeframes, we did not retrieve data about the use of methylprednisolone intravenous pulses precluding any comparison about this practice. In addition, although we showed a reduction in GC use in our patients between the two time periods, we did not analyze in this study the potential impact on GC-related adverse events.

To conclude, this real-life study showed a global change in practices over time with a reduction in prescribed GC doses starting from the third month, leading to a reduction in the duration of GC use. We did not observe a more important use of immunosuppressants, but the use of TCZ has progressively supplanted the use of methotrexate. Important practice discrepancies were observed among the treating physicians. A replication of these observations in other centers may be interesting to confirm or challenge the changes and practice variabilities we observed.

## Figures and Tables

**Table 1:** Baseline characteristics, treatments and outcomes of GCA patients followed during the two timeframes (2014–2017 and 2018–2020).

	**2014–2017 (n = 96)**	**2018–2020 (n = 84)**	***p***
**Demographics**			
Female	70 (73)	53 (63)	0.16
Age (years)	71 [52–90]	71 [57–90]	0.73
**Cardiovascular risk factors/events**			
Tobacco use	29 (30)	17 (20)	0.13
Hypertension	50 (52)	41 (49)	0.66
Diabetes mellitus	9 (9)	16 (19)	0.06
Dyslipidemia	37 (39)	29 (35)	0.62
History of stroke	4 (4)	3 (4)	1
History of coronary disease	8 (8)	7 (8)	1
**Clinical Manifestations**			
Body weight, kg	62 [45–104]	66 [46–115]	0.02
Cranial signs	74 (77)	70 (83)	0.30
Ophthalmological signs	26 (27)	25 (30)	0.65
Polymyalgia rheumatica	39 (41)	28 (33)	0.31
**Laboratory tests**			
CRP level, mg/L	89 [3–420]	81 [3–380]	0.83
Hemoglobin level, g/dl	11.3 [7.4–15.8]	11.6 [8.6–15.9]	0.19
**Positive histology**	61/93 (66)	48/76 (63)	0.74
**Vascular imaging**			
Positive halo sign on US	38/75 (51)	28/64 (44)	0.42
Large-vessel imaging <15 days after GC initiation	51/90 (57)	58/81 (72)	0.04
Large-vessel vasculitis on imaging	37/90 (41)	28/81 (35)	0.38
**Glucocorticoids management**			
Introduction dose in mg/kg/day (n = 180)	0.76 [0.25–1.43]	0.72 [0.11–1.3]	0.07
Dose at Month 3 in mg/kg/day (n = 180) *	0.39 [0.14–0.9]	0.29 [0.08–1]	0.002
Dose at Month 6 in mg/kg/day (n = 177) *	0.18 [0.02–0.625]	0.13 [0.04–0.69]	0.0008
Dose at Month 12 in mg/kg/day (n = 162) *	0.10 [0.01–0.83]	0.07 [0.01–0.34]	0.01
Dose at Month 18 in mg/kg/day (n = 125) *	0.09 [0.0001–0.5]	0.08 [0.02–0.39]	0.83
Dose at Month 24 in mg/kg/day (n = 93) *	0.08 [0.01–0.67]	0.06 [0.01–0.24]	0.02
Patients who stopped GC at <12 months	5 (5)	13 (15)	0.03
Patients who stopped GC at <18 months	20 (21)	35 (42)	0.003
Patients who stopped GC at <24 months	39 (41)	48 (57)	0.03
**Use of an immunosuppressant**	30 (31)	32 (38)	0.34
Methotrexate	19/30 (63)	12/32 (37)	0.04
Tocilizumab	11/30 (37)	20/32 (63)	0.04
**Outcomes**			
Total follow up, months	50 [24–98]	25 [24–57]	<0.0001
Relapse	55 (57)	45 (54)	0.62
Death after 24 months	6 (6)	2 (2)	0.29

Numbers are values (%) or medians [ranges]. CRP—C-reactive protein; GC—Glucocorticoids; US—ultrasonography. * in patients still under GC treatment.

**Table 2:** Significant differences in GCA management among the 7 physicians.

	Ranges among Physicians	*p*
**Performance of large-vessel imaging <15 days after GC initiation**	48 to 72%	0.04
**GC dose at Month 6**		
in mg/day	Medians: 8 to 16 mg/day	0.008
in mg/kg/day	Medians: 0.10 to 0.25 mg/kg/day	0.004
**GC dose at Month 12**		
in mg/day	Medians: 3 to 6.5 mg/day	0.03
in mg/kg/day	Medians: 0.06 to 0.10 mg/kg/day	0.04
**Total GC duration in those who stopped GC, months**	Medians: 15 to 34 months	0.02
**GC stopped at <18 months**	0 to 29%	0.007

GC—glucocorticoids.

**Table 3:** GCA characteristics and therapeutic management according to the different physicians in the department.

	P1			P2			P3			P4	P5	P6	P7
	2014–2017	2018–2020	*p*	2014–2017	2018–2020	*p*	2014–2017	2018–2020	*p*	2014–2017		2017–2020	
**Vasculitis demonstration**													
Positive temporal artery biopsy	68%	50%	0.21	50%	64%	0.67	70%	50%	0.40	82%	53%	71%	77%
Large-vessel vasculitis	57%	50%	0.78	40%	27%	0.66	30%	0	0.14	45%	44%	25%	29%
**GC management**													
Dose at introduction, mg/day	60 [35–80]	50 [10–100]	0.20	40 [30–70]	45 [40–65]	0.51	50 [35–70]	50 [40–80]	0.16	50 [40–80]	50 [20–80]	50 [35–80]	60 [20–85]
Dose at Month 3, mg/day	20 [7–60]	20 [7–60]	0.38	30 [12.5–40]	25 [10–60]	1	25 [10–40]	17.5 [10–40]	0.23	22.5 [15–45]	25 [10–50]	20 [10–45]	19 [12.5–40]
Dose at Month 6, mg/day	10 [5–30]	10 [5–40]	0.68	15 [8–30]	13.25 [8–25]	0.65	10 [6–20]	8.5 [6–40]	0.20	10 [6–25]	16 [1–30]	8 [2–25]	9 [5–40]
Dose at Month 12, mg/day	5.5 [1–17.5]	5 [2–20]	0.46	5 [2–18]	8.5 [2–20]	0.41	6 [2–20]	4.5 [2–10]	0.44	5.5 [3–10]	8 [3–40]	3.5 [1–7]	6 [1–12.5]
GC stopped at <12 months	12%	12%	1	0	0	-	0	33%	0.02	8%	0	29%	0
GC stopped at <18 months	30%	40%	0.58	22%	10%	0.58	10%	67%	0.004	25%	15%	71%	14%
GC stopped at <24 months	45%	56%	0.43	22%	20%	1	25%	78%	0.01	67%	35%	82%	43%
GC duration in those who stopped	21 [8–86]	15 [6–40]	**0.04**	37 [14–65]	36.5 [13–49]	0.77	30 [12–62]	14.5 [5–22]	0.002	18.5 [10–48]	24 [14–90]	15 [10–41]	20 [12–41]
Discontinuation at the last FU	88%	72%	0.18	78%	80%	1	80%	78%	1	83%	80%	88%	71%
**Immunosuppressant use**	42%	52%	0.47	33%	50%	0.65	35%	33%	1	16%	15%	30%	36%
Methotrexate	30%	16%	0.24	22%	20%	1	15%	11%	1	8%	10%	6%	29%
Tocilizumab	12%	36%	**0.048**	11%	30%	0.58	15%	22%	0.63	8%	5%	24%	7%
**Antiplatelets**	64%	40%	0.07	78%	40%	0.1	75%	89%	0.63	83%	75%	53%	64%
**Relapse**	64%	68%	0.79	44%	80%	0.17	80%	44%	0.09	25%	55%	35%	50%
**Death**	6%	4%	1	11%	0	0.47	5%	11%	1	0	10%	0	0

Numbers are percentages or medians [ranges]. P—physician; GC—glucocorticoids; FU—follow up.

## Data Availability

Data are available by request to the corresponding author.
